# Possible Role of 1-Aminocyclopropane-1-Carboxylate (ACC) Deaminase Activity of *Sinorhizobium* sp. BL3 on Symbiosis with Mung Bean and Determinate Nodule Senescence

**DOI:** 10.1264/jsme2.ME15120

**Published:** 2015-12-09

**Authors:** Panlada Tittabutr, Sudarat Sripakdi, Nantakorn Boonkerd, Waraporn Tanthanuch, Kiwamu Minamisawa, Neung Teaumroong

**Affiliations:** 1 School of Biotechnology, Institute of Agricultural Technology, Suranaree University of TechnologyNakhon Ratchasima, 30000Thailand; 2 Synchrotron Light Research Institute (public organization)Nakhon Ratchasima 30000Thailand; 3 Graduate School of Life Sciences, Tohoku University2–1–1 Katahira, Aoba-ku, Sendai, Miyagi 980–8577Japan

**Keywords:** ACC deaminase, determinate nodule, competitiveness, mung bean, nodule senescence

## Abstract

*Sinorhizobium* sp. BL3 forms symbiotic interactions with mung bean (*Vigna radiata*) and contains *lrpL*-*acdS* genes, which encode the 1-aminocyclopropane-1-carboxylate (ACC) deaminase enzyme that cleaves ACC, a precursor of plant ethylene synthesis. Since ethylene interferes with nodule formation in some legumes and plays a role in senescence in plant cells, BL3-enhancing ACC deaminase activity (BL3^+^) and defective mutant (BL3^−^) strains were constructed in order to investigate the effects of this enzyme on symbiosis and nodule senescence. Nodulation competitiveness was weaker in BL3^−^ than in the wild-type, but was stronger in BL3^+^. The inoculation of BL3^−^ into mung bean resulted in less plant growth, a lower nodule dry weight, and smaller nodule number than those in the wild-type, whereas the inoculation of BL3^+^ had no marked effects. However, similar nitrogenase activity was observed with all treatments; it was strongly detected 3 weeks after the inoculation and gradually declined with time, indicating senescence. The rate of plant nodulation by BL3^+^ increased in a time-dependent manner. Nodules occupied by BL3^−^ formed smaller symbiosomes, and bacteroid degradation was more prominent than that in the wild-type 7 weeks after the inoculation. Changes in biochemical molecules during nodulation were tracked by Fourier Transform Infrared (FT-IR) microspectroscopy, and the results obtained confirmed that aging processes differed in nodules occupied by BL3 and BL3^−^. This is the first study to show the possible role of ACC deaminase activity in senescence in determinate nodules. Our results suggest that an increase in ACC deaminase activity in this strain does not extend the lifespan of nodules, whereas the lack of this activity may accelerate nodule senescence.

*Rhizobium*-legume symbiotic interactions benefit agriculture due to the fixation of atmospheric nitrogen gas and its conversion into the plant usable form of ammonia, which ultimately reduces the application of chemical fertilizers to legumes. Infectivity, competitiveness, and nitrogen fixation efficiency are important for achieving improvements in the efficiency of rhizobial inoculation. A strategy using the enzyme 1-aminocyclopropane-1-carboxylate (ACC) deaminase has recently been employed in order to improve plant growth-promoting rhizobacteria (PGPR) biofertilizers. PGPR containing this enzyme cleave ACC, the precursor of ethylene biosynthesis, which may be exuded from plants, to ammonia and α-ketobutyrate instead of its conversion to ethylene. This decreases the amount of ACC, which reduces endogenous ethylene levels in plant cells, thereby promoting plant growth, particularly under stress conditions (10, 11, 22, 32, 38).

In *Rhizobium*-legume symbiotic interactions, ethylene is known to play a role in different stages of the nodulation process. Oldroyd *et al.* (29) previously demonstrated that ethylene inhibited the expression of nod factor (NF)-dependent *rip1* and *ENOD11* genes in *Medicago trancatula* and also disturbed calcium spiking signals. The number of root hair cells that spiked in response to NF was also significantly lower in plants exposed to ACC than in plants exposed to L-α-(2-aminoethoxyvinyl) glycine (AVG), an ethylene inhibitor in synthesis and perception. Moreover, ethylene is also involved in the formation and positioning of root nodule primordia. Nodule primordia typically develop in the root cortex opposite the protoxylem poles, while ethylene is produced in the phloem poles and may interfere with the formation of primordia (14). The *sickle* mutant (an ethylene-insensitive line) of *M. trancatula* was previously shown to form nodules with the same frequency throughout all parts of the root, and not only at the protoxylem poles (31). Furthermore, the number of primordia formed was found to be increased in the *Lotus japonicus etr1-1* mutant line, which contains a mutated ethylene receptor (13). Previous studies have also shown that the root nodule primordium number was increased by the addition of AVG, whereas ACC reduced the number of nodules, suggesting that ethylene controls the formation of nodule primordia and, subsequently, the successful formation of root nodules (28, 53). Thus, the benefit of using rhizobia with ACC deaminase activity to enhance nodulation has been demonstrated in a large number of legumes (10, 11, 16, 18, 27, 32, 43). However, *Glycine max* (soybean) does not exhibit ethylene-inhibited nodulation (36).

Ethylene is not only involved in the nodulation process of legumes, but also plays a role in senescence. The *sickle* mutant line of *M. truncatula* also showed phenotypes such as delayed petal and leaf senescence and reduced seedpod and leaf abscission (30). Moreover, the transformation of the mutated ethylene receptor gene, *Cm-ERS1/H70A* from *Cucumis melo*, into *L. japonicus* reduced ethylene sensitivity, induced petal senescence, and delayed detachment more than those in wild-type plants (28). Moreover, suppressing the production of ethylene has been suggested to assist in nodule maintenance by delaying senescence because ethylene typically participates in senescence in plant cells. Therefore, strong ACC deaminase activity in bacteroids may delay nodule senescence by reducing ethylene production and, as a consequence, prolong nitrogen fixation, which may represent another strategy to improve the efficiency of rhizobial inoculation. A previous study indicated that the enhancement of ACC deaminase activities in *Rhizobium* sp. TAL1145 bacteroids by carrying multiple copies of the *acdS* gene, which encodes the structural component of the enzyme derived from *Sinorhizobium* sp. BL3, inside *Leucaena leucocephala* nodules increased the number of nodules, nodule dry weight, and root dry weight after 16 weeks, but not at an earlier stage. The effects of bacteroids containing ACC deaminase on the growth of leguminous trees may be small, but cumulative over a long period of growing time (43). Nevertheless, the nodules formed by rhizobia in leucaena are the indeterminate type, which continue to grow longitudinally and produce new bacteroid zones for nitrogen fixation. Thus, although difficulties are associated with evaluating the effects of ACC deaminase activity on nodule senescence, they may be clarified in experiments using determinate nodules.

In the present study, the role of ACC deaminase activity on symbiosis and nodule senescence was evaluated in *Vigna radiata* (mung bean), a tropical legume that forms determinate nodules and in which nodulation is affected by alterations in plant ethylene levels (8, 44), inoculated with *Sinorhizobium* sp. BL3 and its derivatives defective in ACC deaminase activity (BL3^−^) and with enhanced enzyme activity (BL3^+^). The relationship between ACC deaminase activity produced by bacteroids inside nodules and their roles in symbiosis and nodule senescence was observed periodically using basic plant growth determinations, and also Fourier Transform Infrared (FT-IR) spectroscopy to verify biochemical changes inside nodules. The effects of ACC deaminase activity on nodulation competitiveness was also investigated in the present study.

## Materials and Methods

### Bacterial culture conditions

*Sinorhizobium* sp. strain BL3 (43) was maintained in yeast extract mannitol (YEM) medium (40). In order to determine the growth of cells in ACC containing medium, BL3 and its derivatives were cultured in YEM broth at 28°C, 200 rpm, with shaking for 3 d prior to transferring to minimal medium (15) supplemented with 10% (v/v) YEM broth. ACC (Sigma-Aldrich, USA) was added to minimal medium at various concentrations from 1–3 mM according to the purpose of each experiment.

### Detection of copy numbers of *lrpL* and *acdS* genes using Southern blot hybridization

In order to determine the copy numbers of the *lrpL* and *acdS* genes in the genome of BL3, genomic DNA was extracted and digested with *Bam*HI and *Eco*RI prior to an analysis with electrophoresis using a 1% (w/v) agarose gel. Southern blot hybridization was performed following the standard method (35). The 1.6-kb DNA fragment containing the *lrpL* and *acdS* genes was used as a probe and labeled with digoxigenin (DIG high-prime DNA labeling kit; Roche Diagnostics, Switzerland) for detection.

### Construction of the BL3 transconjugant containing multiple copies of *lrpL* and *acdS*

**Fig. 1:**
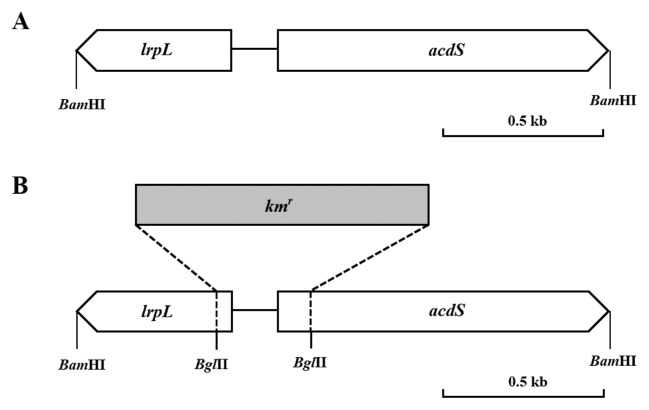
Organization of *lrpL* and *acdS* genes in *Sinorhizobium* sp. BL3 (A), and mutagenesis of *lrpL* and *acdS* by insertion of the kanamycin-resistant gene, *Km^r^* (B) into the chromosome of BL3.

The *lrpL* and *acdS* genes (Accession no. EU183545) were PCR amplified from BL3 genomic DNA as one fragment using the primer BL3-ACCF3-Bam (5′-CAAAGGATCCCTAGAAGGGCAACCTCGCGTTCTC-3′) and Sm-ACCR3-Bam (5′-CGCAGGATCCTCAGCCGTCCCTGTAGTAATAGCT-3′) and cloned into pRK404 (7), a multiple copies plasmid (4–7 copies) at the *Bam*HI site in order to obtain pRK404::*lrpLacdS*. The nucleotide sequences of *lrpL* and *acdS* from BL3 were determined by Macrogen (Seoul, South Korea), and the sequences obtained were confirmed to match the 1.6-kb fragment of BL3 containing the *lrpL* and *acdS* genes as previously described (43). The plasmid pRK404::*lrpLacdS* was transferred into BL3 using the triparental mating method to obtain BL3 containing multiple copies of the *lrpL* and *acdS* genes (BL3^+^ strain) (Fig. 1A).

### Construction of the *lrpL-acdS* mutant of BL3

Gene deletion mutagenesis was performed by replacing the 0.9-kb kanamycin resistance (*km^r^*) gene in the *Bgl*II site of the DNA region containing the *lrpL* and *acdS* genes on pRK404::*lrpLacdS*, which resulted in 54 bp of the *lrpL* gene, 95 bp of the *acdS* gene, and a part of the 143-bp promoter region being removed (Fig. 1B). The constructed plasmid was transferred into BL3 using biparental mating. The mutant was created by double homologous recombination (41) using the incompatible plasmid pHIJI (Gen^r^) to eliminate the other P1-group recombinant plasmid (4). The mutant (BL3^−^ strain) was selected from colonies that were resistant to kanamycin and gentamycin, but sensitive to tetracycline.

### Construction of GUS-labeled BL3

*Escherichia coli* strain S17-1 harboring the plasmid pCAM120 (52) was used for biparental mating with BL3. The blue transconjugant of GUS-labeled BL3 expressing GUS activity was selected on YEM agar containing 20 μg mL^−1^ spectinomycin, 100 μg mL^−1^ streptomycin, and 0.5 mg mL^−1^ 5-bromo-4-chloro-3-indolyl glucuronide (X-gluc dissolved in dimethyl formamide at 25 mg mL^−1^) (39).

### Nodulation competition assay

The germinated seeds of *Vigna radiata* cultivar SUT1 were grown in Leonard jars. Two-day-old seedlings were inoculated with 1 mL of a mixed inoculum containing two strains of BL3 with BL3^+^ and BL3 with BL3^−^ at three different ratios: 1:1, 1:0.5, and 0.5:1 of 10^8^ cells per seed. Three replicates of plants were inoculated with each of the bacterial mixtures and all nodules were collected from each treatment 4 weeks after the inoculation. Each cleaned root nodule was cut in half and GUS staining was performed as described previously (20). The nodule occupancy of each bacterium in the mixture was calculated as a proportion based on the results of GUS staining of all collected nodules in each treatment. However, it should be noted that only the wild-type strain was transformed to contain the GUS marker. Any nodule infected with a mixed population of strains was classified as being occupied by the BL3 wild-type strain. Therefore, the frequency of BL3^−^ and BL3^+^ strains may have been underestimated in this experiment.

### ACC deaminase activity assay

Bacterial cells were grown in 15 mL YEM medium at 28°C, 200 rpm, for 4 d until cells reached the early stationary phase. Cells were washed twice with minimal medium and resuspended in 15 mL of YEM-supplemented minimal medium containing 1 mM ACC, followed by an incubation at 28°C, 200 rpm, for 40 h to induce the production of the ACC deaminase enzyme. ACC deaminase activity was determined by measuring the production of α-ketobutyrate as described previously (5, 15, 18).

### Relative nodulation rate

*V. radiata* cv. SUT1 seeds were surface sterilized and grown in plastic growth pouches filled with N-free plant nutrient solution (40). The 5-d-old rhizobial cultures of each treatment, at a cell concentration of 10^8^ cells mL^−1^, were inoculated into the 2-d-old seedlings of plants in triplicate for each treatment. Data of old and young nodule number accumulation in the same pouch were counted in each treatment from the 2^nd^, 4^th^, and 6^th^ week after the inoculation. The nodulation rates on the 4^th^ and 6^th^ weeks were calculated relative to the number of nodules on the 2^nd^ week after the inoculation.

### Plant experiments

*V. radiata* cv. SUT1 seeds were surface sterilized, germinated, and grown in modified Leonard’s jars containing sterilized vermiculite. The 5-d-old rhizobial cultures of all treatments: BL3, BL3^+^, and BL3^−^ at a cell concentration of 10^8^ cells mL^−1^, were inoculated into 2-d-old seedlings and compared to plants inoculated with the commercial mung bean inoculant *Bradyrhizobium* sp. PRC008 (44) under the same conditions. A sterilized nitrogen-free plant nutrient solution (40) was applied to the plants being tested, which were then grown under cool white fluorescent light (approximately 200 μmol m^−2^ s^−1^), 25°C, and a 12-h/12-h light/dark cycle. The experiment was arranged in a completely randomized design (CRD) with 3 replications. Plants were sampled on the 3^rd^, 5^th^, and 7^th^ week after the inoculation. Plants and nodules were characterized according to the methods described below.

### (i) Determination of nitrogenase activity

Plants were incubated in rubber-capped plastic jars with a 30-mL capacity, and 5% (v/v) of the air was withdrawn from the incubation vessels and replaced with acetylene. These vessels were incubated at 25°C for 30 min. One mL of the gas sample was injected into gas chromatography (GC) by using PE-alumina capillary column filled with Al_2_O_3_ (Perkin Elmer, USA) with 150°C of injector, 200°C of oven, and 50°C of Flame Ionization Detector (FID) (40).

### (ii) Determination of plant biomass, nodule number, and nodule dry weight

Immediately after the determination of nitrogenase activity, shoots were removed from whole plants at the cotyledon leaf scar in order to separate shoots from roots. The number of nodules was counted and nodule, shoot, and root dry weights were then measured by drying the samples at 70°C for 48 h before weighing.

### (iii) Microscopic observations

Infected nodules were separately collected and cleaned using sterilized water before 50-μm-thick cross-sections were cut using a vibratome (Microm HM650V). Nodule samples were stained using 0.05% (w/v) toluidine blue solution, and bacteroid morphology and nodule senescence were subsequently observed under a compound microscope (46).

### (iv) Determination of *acdS* gene expression and ACC deaminase activity in nodules

One gram of root nodules was harvested and ground into a fine powder using a mortar cooled with liquid nitrogen. The powder was suspended in 200 μL of deionized water and subsequently centrifuged at 2,000 rpm for 5 min. The supernatant was transferred into a new tube and bacteroids were sedimented by centrifugation at 2,000 rpm for 2 min. RNA from the bacteroid pellet was extracted using the RNeasy Mini kit (Qiagen, Netherlands). In order to construct cDNA, RT-PCR was performed using the cDNA synthesis kit, SuperScript^®^ (Invitrogen, USA). PCR was conducted using a primer specific to the *acdS* of BL3 in a 25-μL reaction under the conditions of 95°C for 2 min and 30 cycles of 95°C for 30 s, 55°C for 30 s, and 72°C for 1 min. The PCR products were run on an agarose gel and stained with ethidium bromide before visualizing with UV illumination. The expression of genes was normalized to that of the 16S rRNA gene at each time point. The bacteroid pellet was also separated and ACC deaminase activity was measured using the same method as described above.

### (v) Tracking changes in biochemical molecules during the nodulation period using FT-IR microspectroscopy

Four-micrometer-thick cross-sections of nodule samples on the 3^rd^, 5^th^, and 7^th^ week after the inoculation were cut using a vibratome (Microm HM650V). Sections were deposited onto IR reflective slides (Kelvey Mirr-IR slides; Tienta Technology, Ohio, USA), and then air-dried under a vacuum (~1 atm<ambient) in a desiccator for at least 2 d prior to being analyzed. FTIR measurements were conducted at an offline IR spectroscopy facility at the Synchrotron Light Research Institute (a Public Organization), Thailand. Absorption spectra were acquired using a Bruker Vertex 70-IR spectrometer coupled with an IR microscope (Hyperion 2000) and connected with an MCT (HgCdTe) detector cooled with liquid nitrogen over the reflection mode; 64 scans, an aperture size of 68 mm×68 mm, resolution of 6 cm^−1^, and measurement range of 4,000–600 cm^−1^. Spectral acquisition and instrument control were performed using OPUS 6.5 software (Bruker Optics Ltd, Ettlingen, Germany). At least 120 spectra were obtained from each treatment. Spectra from each treatment were analyzed using a Principal Component Analysis (PCA) (2). Spectra were preprocessed by taking the second derivative using the Savitzky-Golay algorithm with nine points of smoothing and normalized with Extended Multiplicative Signal Correction (EMSC) using Unscrambler software (version 9.7, CAMO Software AS, Oslo, Norway), employing the combined spectral ranges of 3,000–2,800 cm^−1^ and 1,800–700 cm^−1^. PCA was performed on each treatment in order to select high quality spectra for further combined analysis. Spectra were rejected (<5% of the total number acquired) on the basis of high residual variance across the first six PCs.

## Results

### Increasing copy number and interruption of *lrpL-acdS* genes in *Sinorhizobium* sp. BL3 and their effects on ACC deaminase activity in the free-living state

The results of Southern blot hybridization revealed the presence of a single copy of the *acdS* gene encoding the structural protein of the ACC deaminase enzyme in the genome of *Sinorhizobium* strain BL3 (Fig. S1). In order to investigate the effects of the ACC deaminase activity of BL3 on symbiosis with mung bean and nodule senescence, BL3 containing the plasmid pRK404::*lrpLacdS* was constructed in order to produce 4–7 copies of these genes in the genome (BL3^+^), while the *lrpL* and *acdS* genes in the chromosome were interrupted in order to create a malfunction in the ACC deaminase enzyme in strain BL3 (BL3^−^). The ACC deaminase activities of these constructs were significantly different from that of the BL3 wild-type in the free-living state (Table 1). ACC deaminase activity was 3.6-fold higher in strain BL3^+^ than in the wild-type, whereas that of *lrpLacdS* mutant strain BL3^−^, although present, was markedly lower than that of the wild-type. These constructs were used to further investigate the effects of rhizobial ACC deaminase on nodulation competition, symbiosis, and nodule senescence.

### Requirement of ACC deaminase activity in *Sinorhizobium* BL3 for nodulation competitiveness in mung bean

**Fig. 2:**
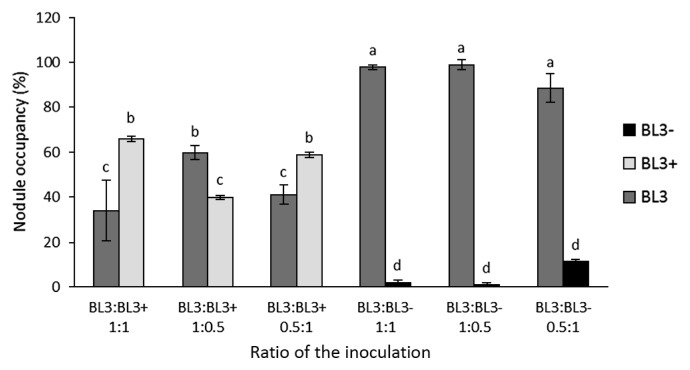
Nodulation competitiveness of BL3^+^ and BL3^−^ when co-inoculated with the BL3 wild-type in mung bean (*V. radiata* cv. SUT1) at different inoculation ratios. Values represent the mean (*n*=3) and error values show the variability in occupancy among triplicate plants for each treatment. Across all treatments, means labeled with different letters are significantly different at *P*<0.05.

Since ethylene interferes with the early stage of the nodulation process, the role of ACC deaminase activity in nodulation competitiveness requires further study. The nodulation competitiveness of strain BL3 with BL3^+^ and BL3^−^ in mung bean was examined at different ratios. We hypothesized that if two strains are equally competitive for nodulation, then the occupancy ratio will vary in proportion to their relative frequencies in an inoculation mixture. At the 1:1 ratio of BL3:BL3^+^, the nodule occupancy of BL3 was 34%, which was significantly lower than that of BL3^+^ (66%). Although the ACC deaminase activity of BL3^+^ was higher than that of the wild-type, the nodule occupancy of BL3^+^ decreased to 40% at the 1:0.5 ratio of BL3:BL3^+^. Meanwhile, competitiveness at the 0.5:1 ratio of BL3:BL3^+^ did not significantly increase the nodule occupancy of BL3^+^ over that at the 1:1 ratio (Fig. 2). The nodule occupancy of BL3^−^ remained at only 2% and was reduced to 1% at the 1:1 and 1:0.5 ratios of BL3:BL3^−^. Furthermore, increasing the cell concentration of BL3^−^ at the 0.5:1 ratio of BL3:BL3^−^ did not overcome competition with the wild-type (Fig. 2). Therefore, the malfunction in the ACC deaminase activity of BL3 clearly affected nodulation competitiveness in mung bean.

### Expression of ACC deaminase in bacteroids during symbiosis with mung bean

**Fig. 3:**
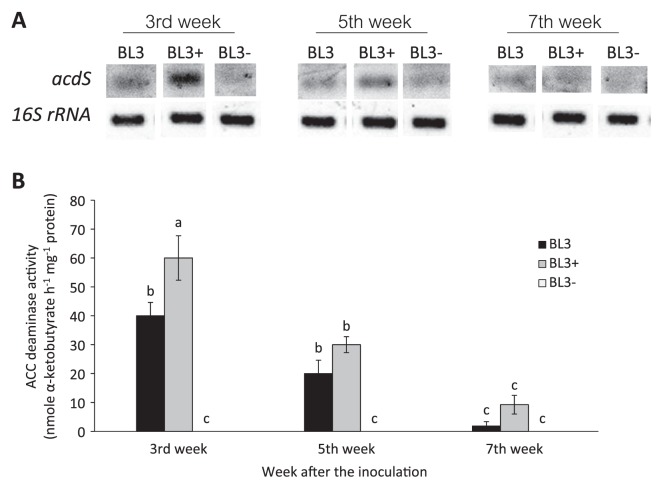
Detection of ACC deaminase expression by RT-PCR of *acdS* and 16S rRNA (A), and ACC deaminase activity (B) from nodules of mung bean (*V. radiata* cv. SUT1) on the 3^rd^, 5^th^, and 7^th^ week after the inoculation with BL3, BL3^+^, and BL3^−^. Values represent the mean (*n*=3) and error values show the variability in measurements of ACC deaminase activity among replicates in each treatment. Across all treatments, means labeled with different letters are significantly different at *P*<0.05.

In order to verify the expression and presence of the ACC deaminase enzyme in the BL3 wild-type and derivative strains (BL3^+^ and BL3^−^) during symbiosis, the expression of *acdS* and ACC deaminase enzyme activity was determined directly from the root nodules of plants on the 3^rd^, 5^th^, and 7^th^ week after the inoculation. ACC deaminase activity was the highest on the 3^rd^ week, and subsequently decreased on the 5^th^ and 7^th^ week after the inoculation, which correlated with gene expression. ACC deaminase activity was not detected inside the nodules of plants inoculated with BL3^−^, but was the highest inside the nodules of plants inoculated with BL3^+^ and it was significantly different from that of BL3 wild-type on the 3^rd^ week after the inoculation. The enzyme activities of BL3 and BL3^+^ were still detected on the 7^th^ week after the inoculation (Fig. 3). These results confirmed the presence of ACC deaminase activity produced by the inoculated strains inside the nodules under a symbiotic state, which may have influenced symbiosis with the plant.

### Effects of ACC deaminase enzyme activity in *Sinorhizobium* BL3 on mung bean growth

**Fig. 4:**
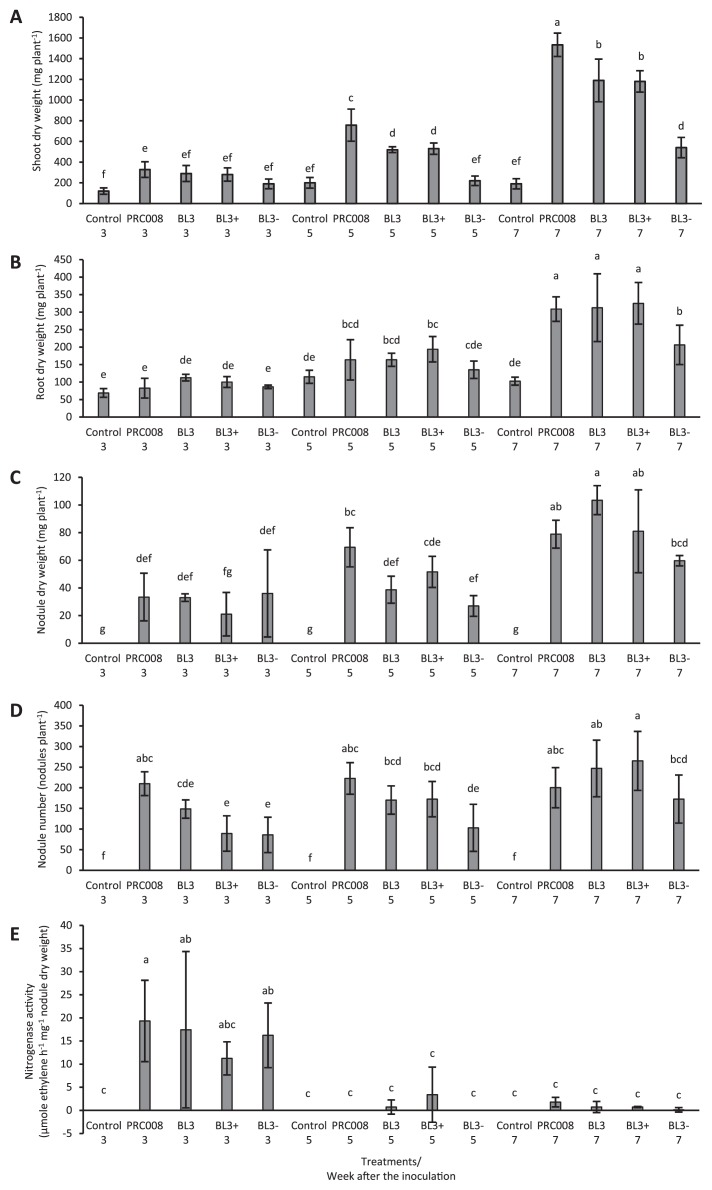
The average shoot dry weight (A), root dry weight (B), nodule dry weight (C), nodule number (D), and nitrogenase activity (E) of mung bean (*V. radiata* cv. SUT1) inoculated with BL3, BL3^+^, and BL3^−^ on the 3^rd^, 5^th^, and 7^th^ week after the inoculation. Values represent the mean (*n*=3) and error values show the variability in measurements among replicates in each treatment. Across all treatments, means labeled with different letters are significantly different at *P*<0.05.

**Fig. 5:**
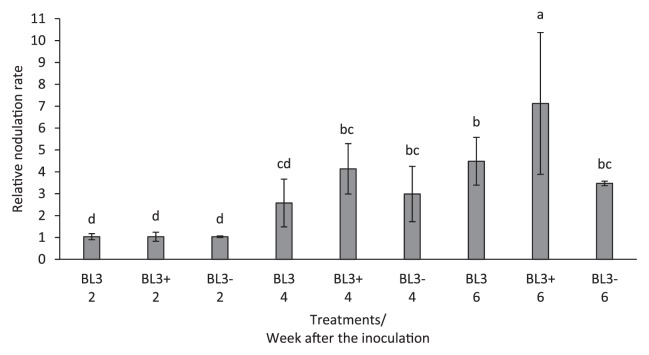
Nodulation rate of mung bean (*V. radiata* cv. SUT1) by BL3, BL3^+^, and BL3^−^ on the 2^nd^, 4^th^, and 6^th^ week after the inoculation. Values represent the mean (*n*=3) and error values show variability in the rate of nodulation among replicates in each treatment. Across all treatments, means labeled with different letters are significantly different at *P*<0.05.

The promotion of symbiosis and plant growth by the BL3 wild-type, BL3^+^, and BL3^−^ strains was investigated under control conditions on the 3^rd^, 5^th^, and 7^th^ week after the inoculation through comparisons with *Bradyrhizobium* sp. PRC008, a commercial mung bean inoculant. The shoot, root, and nodule dry weights as well as the nodule number with all treatments were the highest on the 7^th^ week after the inoculation, while nitrogenase activity was the highest on the 3^rd^ week after the inoculation and then decreased slightly over the course of the planting period (Fig. 4). Shoot dry weight was significantly higher with strain PRC008 than with the BL3 wild-type and derivative strains on the 7^th^ week after the inoculation, whereas no significant differences were observed in nodulation or nitrogen fixation ability between the strains examined. The higher ACC deaminase activity in BL3^+^ did not significantly contribute to the promotion of symbiosis and plant growth more than that in wild-type BL3. However, plant growth and symbiosis on the 5^th^ and 7^th^ week after the inoculation with BL3^−^, which was defective in ACC deaminase activity, were clearly reduced. Moreover, the relative nodulation rate of strain BL3^+^ was significantly increased on the 6^th^ week after the inoculation (Fig. 5). These results indicate that ACC deaminase activity plays some roles in symbiosis between BL3 with mung bean. Nevertheless, the higher ACC deaminase activity in BL3^+^ may have stimulated nodulation at a later state of plant growth, but did not enhance the efficiency of symbiosis or promote the growth of mung bean more than wild-type BL3.

### Role of ACC deaminase activity in mung bean nodule senescence

**Fig. 6:**
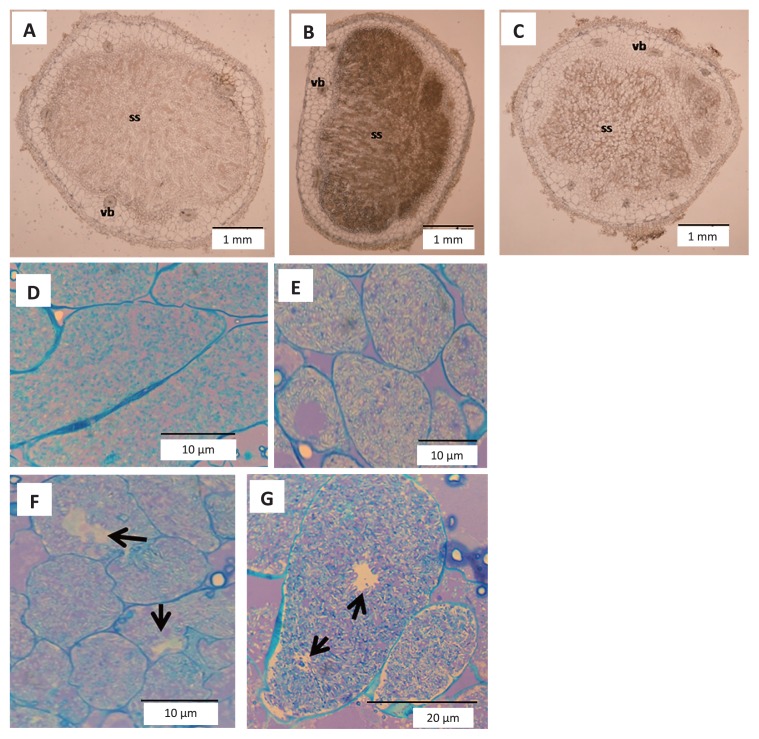
Nodule morphology on the 7^th^ week after the inoculation of mung bean (*V. radiata* cv. SUT1) inoculated with BL3 (A, D), BL3^+^ (B, E), and BL3^−^ (C, F and G) observed under a light microscope at 400× (A, B and C) and 1,000× (D, E, F and G). ss, symbiosome; vb, vascular bundle; the arrow indicates vacuolated cells.

In order to verify the role of the ACC deaminase enzyme on nodule senescence, same aged nodules from plants inoculated with BL3, BL3^+^, and BL3^−^ on the 7^th^ week after the inoculation were examined in thin-layered cross-sections under a light microscope. The results obtained showed that the area of symbiosomes was smaller in nodules occupied by mutant BL3^−^ than in those occupied by BL3 and BL3^+^ (Fig. 6A, B, and C). The transmission electron microscopic assay on nodules on the 7^th^ week after the inoculation revealed fewer bacteroids in the symbiosomes of nodules occupied by BL3^−^ than in those occupied by BL3 and BL3^+^ (Fig. S2). In addition, the toluidine blue staining nodules showing the difference between healthy infected cells and senescent cells (47) were observed in symbiosomes. Nodules occupied by wild-type BL3 showed more blue areas, indicating non-degradable bacteroid cells (Fig. 6D), while dark purple areas represented the degradation of cells, and vacuolated cells (indicated by arrow) were observed along the symbiosomes of nodules occupied by strain BL3^−^ (Fig. 6F and G). This result indicated the degradation of bacteroids inside nodules occupied by ACC deaminase-defective strain BL3^−^, which may enter senescence earlier than nodules occupied by wild-type BL3. Therefore, ACC deaminase activity appears to play a role in maintaining healthy bacteroids during symbiosis. The number of healthy bacteroids was lower in nodules occupied by BL3^+^ than in those occupied by the wild-type strain (Fig. 6E). This result indicated that the increase in ACC deaminase activity in BL3 does not extend the age of nodules, which somehow enter senescence earlier than the wild-type, whereas the lack of ACC deaminase activity appears to induce nodules to enter senescence.

### Tracking changes in biochemical molecules during the nodulation period using data from FT-IR microspectroscopy

The nodules of plants inoculated with BL3, BL3^+^, and BL3^−^ on the 3^rd^, 5^th^, and 7^th^ week after the inoculation were cross-sectioned at a thickness of 4 μm and deposited onto reflection slides. Changes in biochemical molecules in these nodules were tracked using FTIR microspectroscopy. Functional groups of biochemical molecules absorb the specific frequencies of mid-infrared rays that match the transition energy of the vibrating bond or functional group, thereby identifying the specific characteristics of the functional group. For example, lipid bands are detected in the vibration of the C-H stretching region (3,000–2,800 cm^−1^), the amide I (γC=O) and amide II (N-H bend, C-N stretch) regions associated with protein absorbance (1,800–1,500 cm^−1^), and the carbohydrate region assigned mainly to the absorbance of C-O-C asymmetric stretching: cellulose, hemicellulose (Table S1).

**Fig. 7:**
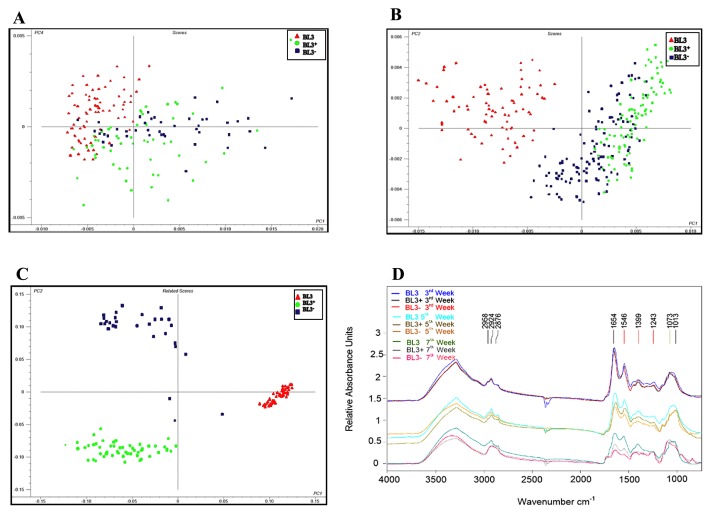
Biochemical component changes in nodules of mung bean (*V. radiata* cv. SUT1). PCA of biochemical components in nodules on the 3^rd^ (A), 5^th^ (B), and 7^th^ week (C), and average spectra on the 3^rd^, 5^th^, and 7^th^ week (D) after the inoculation with BL3, BL3^+^, and BL3^−^.

PCA was selected instead of FTIR spectra in order to distinguish the biochemical profiles of nodules occupied by BL3, BL3^+^, and BL3^−^ with the development of aging. PCA is a multivariate data analysis that reduces the complex variance to the major variance for treatment clustering using linear combinations (*i.e.*, PCs). Thus, information in an entire IR spectrum is reduced to a single point, with coordinates on one, two, or more PCs chosen as axes for plots (2). The shifting of biochemical molecules was clearly demonstrated by PCA, which revealed that most nodule ages influenced changes in biochemical molecules in nodules, whereas rhizobial strains with different ACC deaminase activities also directed the changing of biochemical molecules (Fig. 7). On the 3^rd^ week after the inoculation, the biochemical components inside the nodules of all treatments were similar (Fig. 7A). The patterns of these cellular molecules with each treatment started to differ on the 5^th^ week after the inoculation, and those were separated among nodules occupied by BL3, BL3^+^ and BL3^−^ on the 7^th^ week after the inoculation (Fig. 7B and C).

FTIR is a sensitive technique for detecting conformation changes in the structures of biomolecules. A comparison of the average FTIR absorbance spectrum from each nodulation treatment is shown in Fig. 7D. The FTIR spectral profiles of all treatments were similar on the 3^rd^ week after the inoculation, indicating similar types of biochemical molecules. The protein bands of nodules occupied by BL3^+^ and BL3^−^ were slightly decreased on the 5^th^ week and markedly reduced in nodules on the 7^th^ week after the inoculation when compared with those of wild-type. Changes in biochemical molecules in nodules occupied by each treatment were clearly observed by percent relative of peak integral area analysis (Fig. S3). No significant changes were observed in protein, carbohydrate, or lipid molecules in nodules occupied by BL3 between the 3^rd^, 5^th^, and 7^th^ week, while those occupied by BL3^+^ and BL3^−^ had similar biochemical profiles on the 5^th^ week of nodulation, particularly their protein content, which implies that nodules formed by BL3^−^ and BL3^+^ enter the aging process earlier than those formed by BL3.

Similar results to those for protein components were obtained for lipid components, whereas carbohydrate components increased more in nodules occupied by BL3^+^ and BL3^−^ than in those occupied by the BL3 wild-type on the 7^th^ week after the inoculation. Changes in the levels of the main biochemical molecules in nodules may reveal the hallmark of the nodule senescence stage contributed by rhizobia with different ACC deaminase activities. Taken together, these results indicate that nodules occupied by BL3^−^ enter senescence earlier than those occupied by the wild-type strain, while increases in ACC deaminase activity do not maintain biochemical components inside the nodule which probably indicates the entering into the senescence stage faster than wild-type.

## Discussion

The aim of the present study was to investigate the role of ACC deaminase activity in symbiosis and nodule senescence in mung bean by constructing a *Sinorhizobium* sp. BL3 mutant deficient in ACC deaminase activity (BL3^−^) and enhancing this enzyme activity by transferring a plasmid containing 4–7 copies of its own *lrpL-acdS* genes into BL3 to create BL3^+^. Since only one single copy of the *acdS* gene was found in the genome of *Sinorhizobium* sp. BL3 by Southern blot hybridization, this information ensured the malfunction of the ACC deaminase enzyme after the mutation of the *lrpL-acdS* genes in the chromosome of BL3. Most bacterial genomes in the family *Rhizobiaceae* only contain one copy of *acdS* (26). However, the ACC deaminase activity of mutant strain BL3^−^ was still detected in the free-living state. This result may have been due to the presence of compounds other than α-ketobutyrate having aldehyde or ketone groups that were detectable by the 2,4-dinitrophenylhydrazine reagent in the assay. The ACC deaminase enzyme has the ability to use substrates other than ACC, such as D-serine and D-β-chloroalanine. A previous study showed that the activity of this enzyme was low when D-serine was used as the substrate, and corresponded to approximately 10% of the activity measured with ACC as the substrate (50).

Although Murset *et al.* (23) reported that a malfunction in ACC deaminase activity in the mutant of *B. japonicum* did not impair its ability to nodulate mung bean, the role of ACC deaminase activity in nodulation competiveness by *Sinorhizobium* sp. BL3 in mung bean has been demonstrated. Since mung bean is frequently nodulated by *Bradyrhizobium*, and less so by *Sinorhizobium*, *Sinorhizobium* may need extra tools during the early stages of symbiotic interactions in order to successfully nodulate mung bean, which may not be as well-adapted to interact with this lineage of *Sinorhizobium* as with *Bradyrhizobium*. As shown in Fig. 4, although *Sinorhizobium* sp. BL3 had similar nodulation and nitrogen fixation abilities in mung bean to those of the commercial bradyrhizobial inoculant strain PRC008, the promotion of plant growth by BL3 was still less than that by PRC008. This result implies that interactions between mung bean and *Sinorhizobium* may differ from the mung bean-*Bradyrhizobium* system and may require other mechanisms to increase its compatibility during symbiosis. Ethylene has crucial effects on the different stages of the nodulation process in some legumes (18, 19, 27, 53). Oldroyd *et al.* (29) proposed that the number of infection events increased with decreases in the level of ethylene produced by plant cells because ethylene regulates the NF signal transduction pathway at or upstream of the calcium spiking step and may determine the sensitivity of such legumes to NF produced by rhizobia. Thus, rhizobia that reduce ethylene levels in order to facilitate infection may be beneficial for nodulation competitiveness. The role of ACC deaminase activity in enhancing nodulation competitiveness with alfalfa was also found in *S. meliloti*, which increased ACC deaminase activity (19). Enhancements in nodulation competitiveness may also be caused by increasing root colonization and the number of chances for nodulation. A previous study found that engineered ACC deaminase expressing high levels of enzyme activity in the free-living cells of *Mesorhizobium loti* increased root colonization with *L. japonicus* (6). Moreover, reducing the rate of ethylene biosynthesis by rhizobitoxine, an inhibitor of ACC synthase, also affected nodulation competitiveness. A mutation in the synthesis of rhizobitoxine in *Bradyrhizobium elkanii* has been shown to reduce nodulation competitiveness more than that by the wild-type on siratro (53). These findings highlight the benefit of rhizobia containing tools, such as ACC deaminase or rhizobitoxine, to reduce ethylene levels and promote nodulation competitiveness with the host plant. However, the appropriate levels of these molecules needed to control ethylene levels and support both symbiosis partners have not yet been established.

Although a malfunction in ACC deaminase affected competitiveness, the single inoculation of BL3^−^ formed the same nodule number and promoted plant growth to a similar level as that of wild-type BL3 and BL3^+^ on the 3^rd^ week after the inoculation. This result indicated that rhizobia defective in ACC deaminase still enter plants and form nodules, similar to the wild-type. The level of ethylene in plant cells at the early stage of nodulation may only delay the initiation process of nodulation by BL3^−^, thereby making this strain less competitive for nodulation than the wild-type, which is able to lower the level of ethylene in the early stages of interactions. Nevertheless, plants still accept rhizobia defective in ACC deaminase, and bacteria may slowly enter plant cells and form nodules to fix nitrogen for plant growth. Defective ACC deaminase also did not affect the nitrogenase activity of rhizobia. Since plants may need large amounts of nitrogen during the pre-flowering stage, the nitrogen fixation activity of all strains tested was the highest on the 3^rd^ week after the inoculation. A previous study reported that approximately 28% of the nitrogen content in mung bean was derived from nitrogen fixation at this stage (37). Thus, the effects of ACC deaminase may not be clearly observed at this stage of plant growth, even though this enzyme activity was detected at high levels in nodules occupied by wild-type BL3 and BL3^+^.

The promotion of plant growth by BL3^−^ was significantly reduced on the 5^th^ week after the inoculation. Mung bean entered the flowering stage on the 5^th^ week and the late flowering stage on the 7^th^ week (37). Natural nodule aging is concurrent with a decline in nitrogen fixation in the flowering or pod-filling period. Matamoros *et al.* (21) proposed that some of the proteins necessary for nodule function were already compromised at the mature stage, and this may be the reason for reductions in the rate of nitrogen fixation. However, nodules that maintain nitrogen fixation for a longer period of time have the highest plant dry weights (17). In our study, nitrogenase activity was markedly reduced on the 5^th^ week after the inoculation, which indicated that nodules started to enter senescence (1). Nascimento *et al.* (25) reported that nitrogenase activity in *Mesorhizobium* LMS-1 was more prominent in nodules occupied by a transconjugant that increased ACC deaminase activity (LMS-1 [pRKACC]) than in nodules occupied by the wild-type 31 d after the inoculation, and then decreased to a similar level 45 d after the inoculation. Although no significant differences were observed in nitrogenase activity among the strains in tested the present study on the 5^th^ week after the inoculation, this activity was approximately 3- and 5-fold higher in nodules occupied by BL3 wild-type and BL3^+^ than in nodules occupied by BL3^−^, and this amount of fixed nitrogen may still have contributed to plant growth. The number of nodules and nodule dry weight of plants inoculated with wild-type and BL3^+^ started to increase from the 5^th^ week after the inoculation and were significantly greater than those of BL3^−^ on the 7^th^ week after the inoculation. Moreover, increasing the relative nodulation rate in plants nodulated by BL3^+^ from the 4^th^ and 6^th^ week after the inoculation revealed the role of ACC deaminase activity in facilitating the new establishment of nodules, even in plants that had entered senescence. Similar phenomena were also detected in other rhizobia. *acdS*-defective *M. loti* MAFF030399 had a significantly 3-fold lower number of nodules with *L. tenuis* than those inoculated with the wild type, while the nodulation rate of the strain that increased ACC deaminase activity was approximately 11-fold higher 60 d than 28 d after the inoculation (6). In *Mesorhizobium* LMS-1 wild-type or LMS-1 (pRKACC), which increase ACC deaminase activity, no significant differences were noted in the nodule number formed on chickpea 31 d after the inoculation, while the nodule number of LMS-1 (pRKACC) was significantly higher (2.5-fold) than that of the wild-type 45 d after the inoculation (25). These findings may again confirm the role of ACC deaminase activity in reducing ethylene emissions from plant cells and extend nodule formation to a later stage of plant growth. Thus, the higher number of nodules formed by the BL3 wild-type and BL3^+^ may participate in nitrogen fixation and contribute nitrogen to plant growth, resulting in a significant increase in shoot dry weights at a later stage, whereas the ability of the BL3^−^ strain to form new nodules was reduced and the nodule number was low when the plant entered the flowering stage.

A microscopic analysis of nodules with the same age on the 7^th^ week after the inoculation revealed a role for ACC deaminase in the establishment of symbiosomes. The incomplete area of symbiosomes in nodules occupied by BL3^−^ indicated some inhibitory effects during nodule development. The findings of several experiments have indicated that ethylene does not have an effect after formation of the nodule primordium, but inhibits the step before or at the point of infection initiation. Previous studies have hypothesized that ethylene affects the stability of NF or other effectors that induce the calcium spiking process in *M. trancatula* symbiosis (9, 29, 30, 51). However, ethylene has also been shown to affect the formation and positioning of root nodule primordia in pea, *M. trancatula*, *L. japonicas*, and *Vicia sativa* subsp. *nigra* (12, 13, 31, 48), whereas it has no effect on nodulation in *Glycine max* (36). Therefore, ethylene may be involved in different stages of nodulation or nodule development depending on each legume species. The incomplete establishment of symbiosomes in mung bean nodulated by BL3^−^ may have resulted from interference by ethylene during nodule development due to the lack of ACC deaminase activity in order to relieve the inhibitory effects of ethylene. Thus, bacteroid differentiation in and cytological analysis of nodules required further study in order to demonstrate the role of the ACC deaminase enzyme during nodule development, which may be involved in facilitating decreases in ethylene-induced plant defensive systems.

Regarding the role of ACC deaminase activity in senescence in nodules, the results of toluidine staining and FT-IR analyses indicate a possible role for ACC deaminase activity in maintaining the health of bacteroids during symbiosis. However, more experiments to determine nodule senescence are needed further verified to ensure the role of ACC deaminase in nodule senescence. In the present study, increases in ACC deaminase activity in BL3^+^ did not retard nodule senescence; however, enzyme activity was still detected at low levels in nodules until the 7^th^ week. The high ACC deaminase activity of BL3^+^ at the early nodulation process may allow for the establishment of symbiosomes and nitrogen fixation, which accelerate the availability of N-metabolites in nodules together with a lower carbon supply from plants and reduced C/N ratio inside nodules. This C/N ratio change may be detected and transduced by an abscisic acid-mediated signaling pathway and followed by the activation of proteolytic activities in the senescent stage (34). This proteolytic activity may accelerate damage to bacteroid cells in nodules occupied by BL3^+^ more than in those occupied by wild-type, resulting in detection of bacteroid degradation as well as protein and lipid degradation inside the nodule more than those of wild-type on the 7^th^ week after the inoculation. The shifting of proteins, lipids, and carbohydrates was clearly demonstrated by PCA, which revealed that the subsequent results of different ACC deaminase activities that may contribute to changes in biochemical molecules in the different stages of nodule aging. The protein and lipid components of nodules were slightly decreased on the 5^th^ week and markedly reduced on the 7^th^ week after the inoculation, while carbohydrate components were increased in nodules that had entered the senescent stage. In senescent nodules, the most abundant nonstructural carbohydrate, trehalose was found at up to 84% while up to one-half of trehalose remained during nodule senescence (24). During senescence, membrane lipids (42), proteins, and nucleic acids (3) are degraded through peroxidation by the free radicals generated by bacteroids, which degrades the peribacteroidal membrane (PBM) during senescence (45, 49). Thus, nodule senescence triggers a wide range of proteolytic activities that cause large-scale protein degradation (33) and lead to the final outcome of cell death in bacteroids and nodule cells.

## Conclusion

We herein revealed the important roles of the ACC deaminase activity of *Sinorhizobium* sp. BL3 in symbiosis with mung bean, a tropical legume that forms determinate nodules. The lack of ACC deaminase activity in this strain clearly affected nodulation competitiveness. This enzyme may also participate in the establishment of symbiosomes and maintenance of healthy bacteroids during the development of nodules. A deficiency in ACC deaminase may prompt nodules to enter senescence earlier than the wild-type, whereas an increase in this enzyme activity does not prolong the shelf-life of nodules or directly increase nitrogenase activity; however, it promotes plant growth by stimulating nodule formation at a later stage of plant development. Therefore, rhizobia that contain appropriate levels of ACC deaminase activity need to be considered for improvements in rhizobial inoculations, which may vary in different legumes.

## Supplementary Material



## Figures and Tables

**Table 1:** Comparison of ACC deaminase activities of strains BL3, BL3^+^, and BL3^−^ under the free-living condition.

Strains	ACC deaminase activity (μmole α-ketobutyrate mg^−1^ protein h^−1^)
BL3	4.47±2.29 b
BL3^+^	16.34±1.08 a
BL3^−^	0.07±0.03 c

Mean values followed by different letters are significantly different at 0.05%.
