# Supplementary material for: Possible Role of 1-Aminocyclopropane-1-Carboxylate (ACC) Deaminase Activity of Sinorhizobium sp. BL3 on Symbiosis with Mung Bean and Determinate Nodule Senescence
Source: Microbes Environ. 2015 Dec 9;30(4):310–20. doi: 10.1264/jsme2.ME15120 (PMC4676554; doi:10.1264/jsme2.ME15120)
Supplement: Supplementary file 1 [file 30_310_s1.pdf]

Table S1 Assignment FTIR band of biochemical molecules

Wavenumber (cm^{-1})	Assignment bands	Reference
3000-2800	CH_2, CH_3 symmetric, asymmetric stretch: mainly lipids, with the little contribution from proteins, carbohydrates, nucleic acids	Socrates 2001
~1720-1744	Ester C=O stretch: lipid, triglycerides, carboxylic acid	Socrates 2001
~ 1700-1600	Mainly γ(C=O) associated with protein as the amide I band	Yu (2008)
~1544	Amide II (protein N-H bend, C-N stretch)	Yu (2008)
~1452	CH_2 Bending: lipids	Socrates (2001)
~ 1240	PO_2- phosphodiester group from Nucleic acid and phospholipid	KacÆuraÂkovaÂ (1999)
~ 1080	PO_2- phosphodiester group from Nucleic acid and phospholipid	KacÆuraÂkovaÂ (1999)
~1133, 1058	C-O-C asymmetric stretching: carbohydrate, cellulose, hemicellulose<br>C–C–O polyester	KacÆuraÂkovaÂ (1999)
~979	P-O Phosphate attached to longer methylene chains	Bellamy (1957)

Reference:

Socrates, G. 2001. Infrared and raman characteristic rroup frequencies. pp. 366.

Yu, P. 2008. Molecular chemistry of plant protein structure at a cellular level by synchrotron-based FTIR spectroscopy: Comparison of yellow (*Brassica rapa*) and brown (*Brassica napus*) canola seed tissues. Infrared Phys. Technol. 51: 473-481.

KacÆuraÂkovaÂ, M., N. Wellner, A. EbringerovaÂ, R. H. Wilson, and P. S. Belton. 1999. Characterization of xylan type polysaccharides and associated cell wall components by FT-IR and FT-Raman spectroscopies. Food Hydrocolloids 13: 35-41.

Bellamy, L.J. 1957. The Infra-red Spectra of Complex Molecules, Wiley, New York.

Supplemented data

Figure S1 Southern blot analysis of the copy number of the *acdS* gene in the chromosome of *Sinorhizobium* sp. BL3. (A) Gel electrophoresis of digested chromosomal DNA with *Bam*HI and *Eco*RI, and (B) hybridization with the *acdS*-specific DNA probe labeled with digoxigenin. Lane 1, DNA ladder marker; Lane 2, PCR product of *lrpL-acdS* genes; Lane 3, undigested chromosomal DNA; Lane 4, *Bam*HI and *Eco*RI-digested chromosomal DNA. Lane 5, PCR product of the *lrpL-acdS* gene; Lane 6, undigested chromosomal DNA; Lane 7, *Bam*HI and *Eco*RI-digested chromosomal DNA.

Figure S2 Nodule morphology on the 7th week after the inoculation of mung bean (*V. radiata* cv. SUT1) inoculated with BL3 (A), BL3^{+} (B), and BL3^{-} (C) observed under a transmission electron microscope.

Figure S3 Percent relative of peak integral area analysis of biochemical molecules changes in the nodules occupied by BL3, BL3^{-}, and BL3^{+} on the 3rd, 5th, and 7th week after the inoculation.

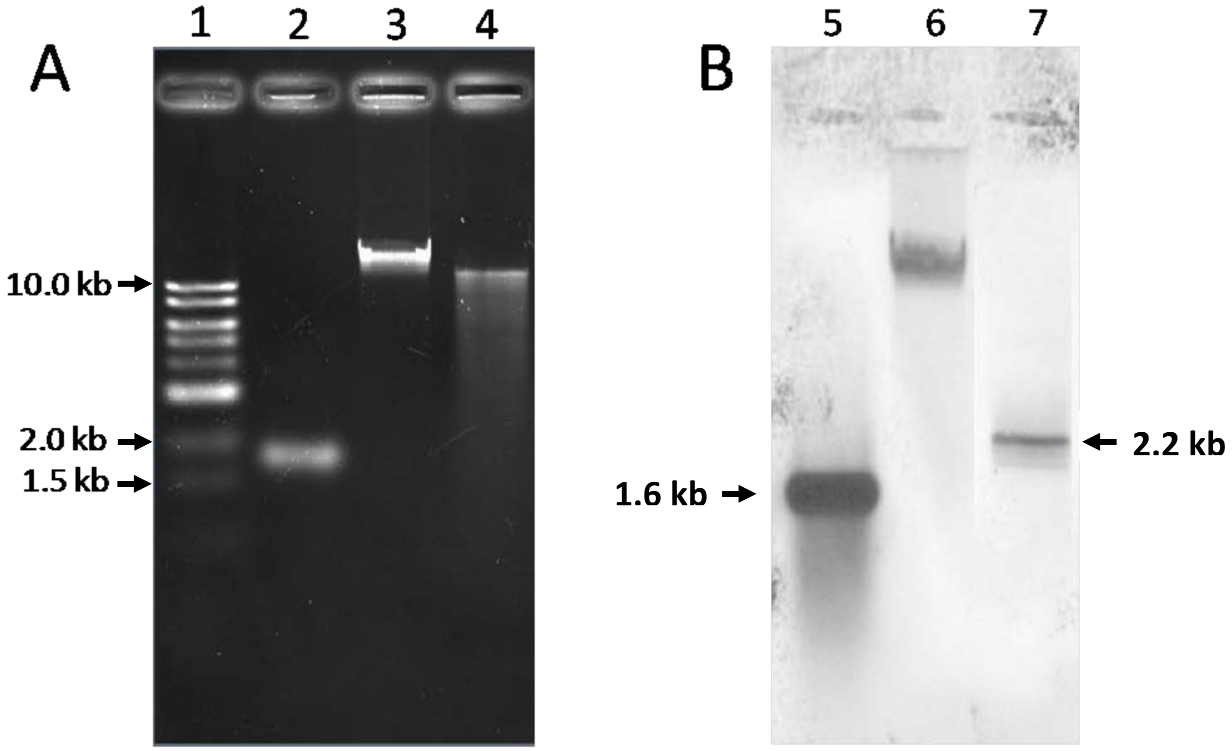
A
B
1
2
3
4
5
6
7
10.0 kb
2.0 kb
1.5 kb
1.6 kb
2.2 kb

A

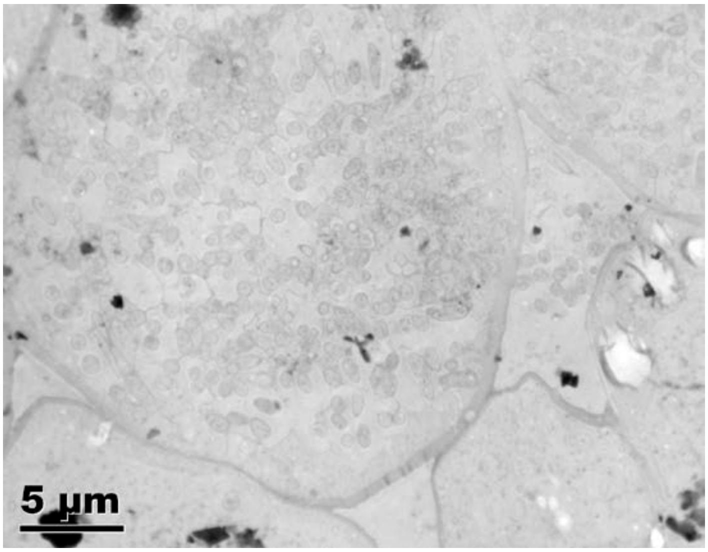

B

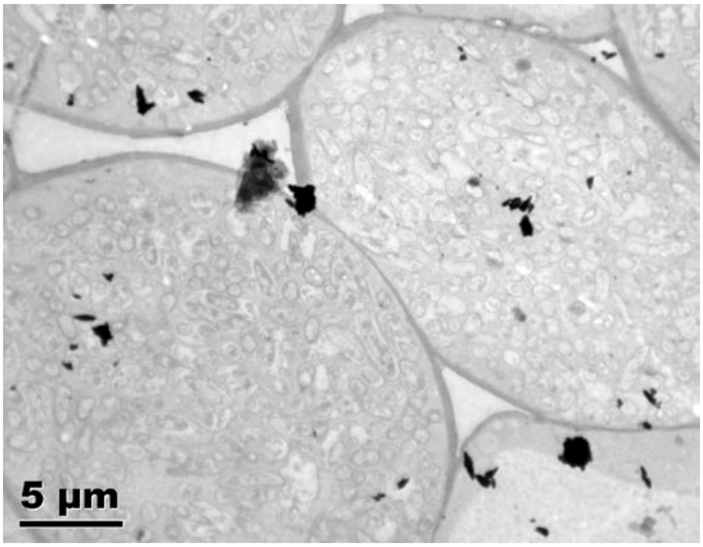

C

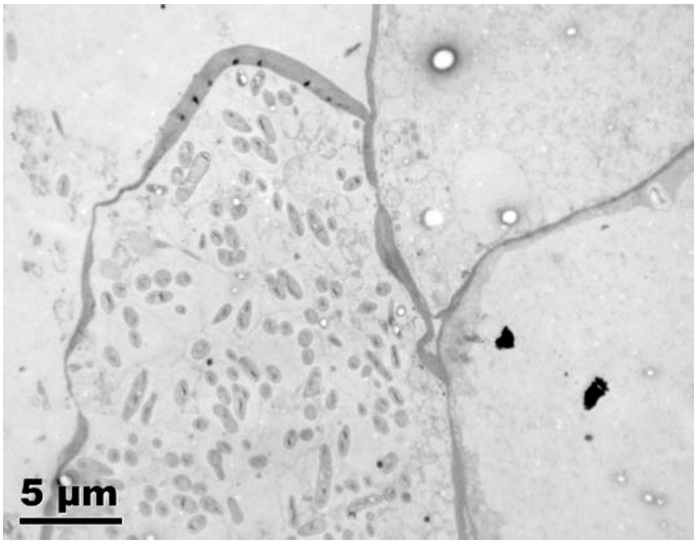

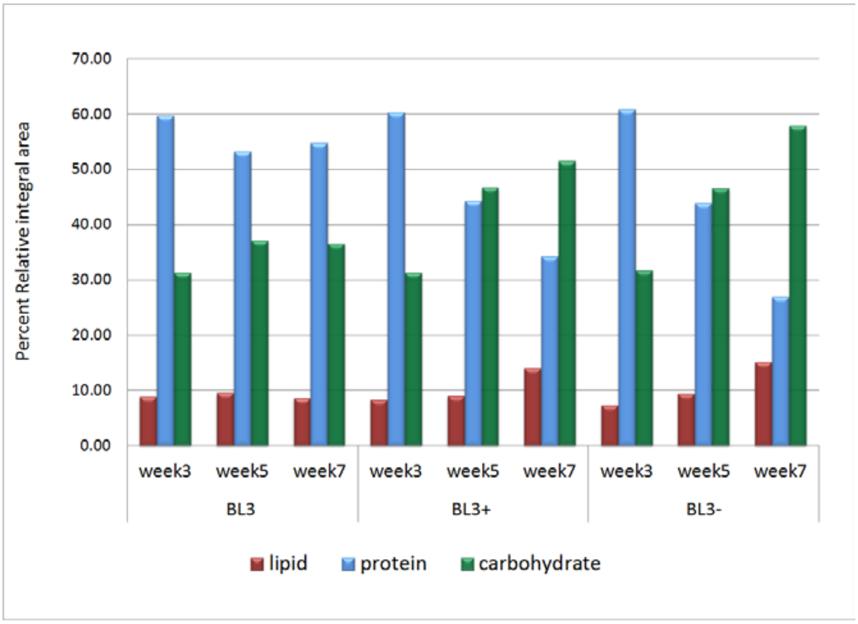

Percent Relative integral area
70.00
60.00
50.00
40.00
30.00
20.00
10.00
0.00
week3
week5
week7
week3
week5
week7
week3
week5
week7
BL3
BL3+
BL3-
lipid
protein
carbohydrate
